# No evidence for the effectiveness of bracing in patients with thoracolumbar fractures: A systematic review

**DOI:** 10.3109/17453670902875245

**Published:** 2009-04-01

**Authors:** Boukje M Giele, Suzanne H Wiertsema, Anita Beelen, Marike van der Schaaf, Cees Lucas, Henk D Been, Jos A M Bramer

**Affiliations:** ^1^Department of RehabilitationAmsterdamthe Netherlands; ^2^Department of Clinical Epidemiology and BiostatisticsAmsterdam the Netherlands; ^3^Department of Orthopaedic Surgery, Academic Medical Center, University of AmsterdamAmsterdamthe Netherlands; ^4^Department of Rehabilitation Medicine, Section of Physical Therapy, VU University Medical CenterAmsterdamthe Netherlands

## Abstract

**Background and purpose** The use of braces is widespread in patients with thoracolumbar fractures. The effectiveness of bracing, however, is controversial. We sought evidence for the effect of bracing in patients with traumatic thoracolumbar fractures based on outcome and length of hospital stay (LOS). Furthermore, we evaluated the incidence of complications of bracing.

**Methods** An electronic search strategy with extensive MeSH headings was used in various databases to identify studies that compared bracing and non-bracing therapies. Two reviewers independently selected systematic reviews, randomized controlled trials (RCTs), controlled clinical trials, and observational studies, and both assessed the methodological quality and extracted the data.

**Results** No systematic reviews or RCTs were found. 7 retrospective studies were included. None of these studies showed an effect of bracing. Because of poor methodological quality, no best-evidence synthesis could be performed. One observational study was selected in which a complication of bracing was reported.

**Interpretation** In the present literature, there is no evidence for the effectiveness of bracing in patients with traumatic thoracolumbar fractures. The lack of high-quality studies prevents relevant conclusions from being drawn.

## Introduction

Currently, bracing is a widely accepted part of nonoperative and operative treatment of patients with thoracolumbar fractures (Dick 1984, White and Panjabi 1990, Hartman et al. 1995, Panjabi et al. 1995, Blauth et al. 1999, Rohlmann et al. 1999, Bakker et al. 2002, Dai 2002, van der Roer et al. 2005). Most commonly, a 3-point corset is used; this is also known as a Jewett brace or extension brace and is thought to prevent rotation and flexion of the spine. The goals of bracing are commonly to prevent failure of osteosynthesis, to facilitate immobilization, and to ensure correct posture (Patwardhan et al. 1990, Connolly and Grob 1998).

There is some controversy in the literature regarding the effectiveness, the necessity, and the possible complications of bracing. Many publications have indicated positive effects, such as relief of pain, reduction of intradiscal pressure, and restriction of gross body motion (Cantor et al. 1993, Cunliffe,1993, Chow et al. 1996, Melchiorre 1999, McNair and Heine 1999, Liu et al. 2003, Tropiano et al. 2003, Pfeifer et al. 2004). Other studies have questioned the necessity of bracing (Benzel and Larson 1989, Ohana et al. 2000, Folman and Gepstein 2003). Axelsson et al. (1992) concluded that external lumbar support has no mechanical stabilizing effect on the lumbar spine and Connolly and Grob (1998) considered that adequate instruction of the patient would have the same effect as bracing. Complications such as skin defects and discomfort after bracing have been reported (Benzel and Larson 1989, McBride 1989, Tezer et al. 2005). Brace therapy may also cause patients emotional distress (Matsunaga et al. 2005).

We performed a systematic review to find evidence for the effect of bracing in patients with traumatic thoracolumbar fractures, non-operatively or operatively treated, on outcomes according to the International Classification of Functioning, Disability and Health (ICF) (WHO 2001) and on length of hospital stay (LOS). In addition, we evaluated the incidence of complications of bracing.

## Methods

### Criteria for consideration of studies for this review

*Types of studies.* Systematic reviews, randomized controlled trials (RCTs) and controlled clinical trials (CCTs) were included. Since we expected to find few of these types of studies, we included also observational studies with a control group (cohort study, case-control). Language was restricted to English, German, French, or Dutch. Only full-length articles were included. To gather as much information as possible, case series were also studied.

*Types of patients.* Studies including adult patients admitted to hospital for traumatic single or multiple, unstable or stable thoracolumbar fracture(s) (T10-L5), followed by nonoperative or operative treatment, were considered. Studies including patients with neurological deficit, fractures due to osteoporosis, and non-traumatic fractures were excluded.

*Types of intervention.* Studies were included that compared patients wearing a brace with patients not wearing a brace. A brace can be defined as a 3-point corset, Jewett brace, or extension brace. Studies involving patients wearing a cast were excluded.

*Types of outcome measures.* We classified the outcomes of interest according to the International Classification of Functioning, Disability and Health (ICF) (WHO, 2001). Studies that included at least one of the following outcome measures were considered: (1) body functions and structure—pain, skin defect, muscle strength, and deformity of the spine; (2) activities—transfers, gait pattern, use of walking aids, walking distance, and activities of daily living (ADL); (3) participation—anxiety, and return to work. Also, quality of life (QOL) and length of hospital stay (LOS) were required as outcome measures.

*Search strategy for identification of studies.* A search was conducted through the following resources: MEDLINE, EMBASE, CENTRAL, CINAHL, the Cochrane Database of Systematic Review (CDSR), and the Database of Abstracts of Reviews of Effects (DARE) (Table 1). All the reference lists of the articles retrieved were examined for additional publications. Studies that appeared potentially relevant were retrieved as a full article. The search was carried out by two reviewers independently (BG, SW).

**Table 1.** Search strategy

Dimension	Location of fracture	Type of fracture	Treatment of fracture	Intervention	Outcome
Search strings	Lumbar$ (TW)	Fracture$ (TW)	Conservative(TW)	3-point corset (TW)	Pain (MH)
	Thoracic$ (TW)	Injury (TW)	Surgical (MH)	3-point corset (TW)	Muscle strength (TW)
	Thoracolumbar (TW)	Trauma$ (TW)	Surgery (MH)	Brace (TW)	Deformity (TW)
	Vertebra$ (TW)	Compression fracture (TW)	Nonoperatively (TW)	Splint (TW)	Transfer (TW)
	Lumbar vertebrae (MH)	Burst fracture (TW)		Corset (TW)	Ambulation (MH)
	Thoracic vertebrae (MH)	Stable fracture (TW)		Extension brace (TW)	Gait pattern (TW)
	Spinal fractures (MH)	Flexion distraction (TW)		Orthosis (TW)	Walking distance (TW)
	Spine (TW)	Fracture dislocation (TW)			Walking aid (TW)
		Unstable fracture (TW)			Skin defect (TW)
					ADL (TW)
					Anxiety (TW)
					QOL (MH)
					LOS (MH)

The dimensions (location of fracture, type of fractire, treatment of fracture, intervention, and outcome) were linked together with AND.

The search strings per dimension were linked together with OR.

TW: text word; MH: mesh heading.

*Study selection.* The reviewers examined the titles and abstracts of the publications identified in order to select studies that met the inclusion criteria. All studies that were considered relevant by at least one of the two reviewers were retrieved. The final inclusion or exclusion was done after examining the full text of potentially relevant articles.

*Quality assessment.* The two reviewers independently assessed the methodological quality of the studies included, with predefined criteria for internal validity of RCTs and CCTs. Inter-reviewer agreement was analyzed, calculating percentage of agreement and a Kappa (κ) score. Cut-off point for inclusion in best-evidence synthesis was defined as 50% of the van Tulder criteria being reached (van Tulder et al. 2003). 2 of these criteria were judged not to be relevant in the case of bracing (blinding of patients and care providers). The decision was thus made that 5 or more items of the van Tulder criteria had to be met. Disagreements in study selection or quality assessment were resolved by discussion. The judgment of a third reviewer (MS) was decisive when disagreement persisted.

*Data extraction and analysis.* We anticipated too much diversity among the studies with regard to the participants (diversity of fractures), interventions (duration, frequency, and setting), and outcome (diversity and presentation of results) to make an appropriate quantitative analysis (meta-analysis). Thus, we used levels of evidence as recommended by the Back Group (van Tulder et al. 2003) to do a qualitative analysis regarding the effectiveness of treatment, taking into account the participants, interventions, controls, outcome measures, and methodological quality of the original studies.

*Incidence of complications.* To obtain data on complications, the above-mentioned strategy was used. No methodological quality assessment of these studies was performed.

## Results

### Study selection

The search strategy resulted in 1,082 references. After selection based on the title and the abstract, 57 full articles were examined. Only 6 of these (Braun et al. 1991, Karjalainen et al. 1991, Shen and Shen 1999, Ohana et al. 2000, Folman and Gepstein 2003, Post et al. 2006) met all the inclusion criteria. 1 additional study (Schlickewei et al. 1991) was retrieved by citation tracking from the studies initially included. Exclusion was based on: study design (35), type of participants (13), or language (2) (Figure). 10 case series, including between 21 and 124 patients, were studied to gather information about the possible effects and complications of bracing and non-bracing (Weitzman 1971, Reid et al. 1988, Blamoutier et al. 1992, Loew et al. 1992, Cantor et al. 1993, Mumford et al. 1993, Chow et al. 1996, Melchiorre 1999, Celebi et al. 2004, Tezer et al. 2005).

**Figure:**
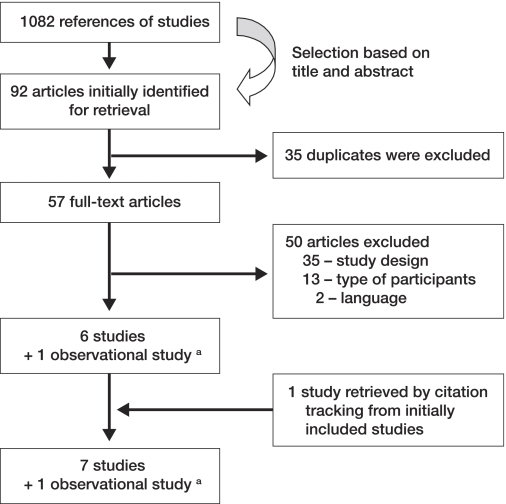
Flow chart of study selection.

### Methodological quality

Initially, there was disagreement between the reviewers (kappa score: 0.59). Most disagreements were resolved at the consensus meeting. The third reviewer had to make a final decision twice. The methodological quality of the studies included was very low (Table 2). None of the studies fulfilled 50% or more of the internal validity criteria. The most prevalent methodological flaws were shortcomings in randomization, treatment allocation, groups at baseline, and compliance.

**Table 2.** Methodological quality of the studies

	List of criteria for internal validity (van Tulder et al. 2003)										
Authors	A	B	C	D	E	F	G	H	I	J	K
Braun et al. (1991)	No	No	No	No	No	No	Yes	?	No	No	No
Folman and Gepstein (2003)	No	No	No	No	No	No	No	?	No	No	No
Karjalainen et al. (1991)	No	No	No	No	No	No	No	?	No	No	No
Ohana et al. (2000)	No	No	No	No	No	No	No	?	No	Yes	No
Post et al. (2006)	No	No	No	No	No	No	No	?	No	No	No
Schlinkewei et al. (1991)	No	No	No	No	No	No	No	?	No	No	No
Shen and Shen (1999)	No	No	No	No	No	No	No	?	No	No	No

A. Was the method of randomization adequate?

B. Was the treatment allocation concealed?

C. Were the groups similar at baseline regarding the most important prognostic factors?

D. Was the patient blinded to the intervention?

E. Was the care provider blinded to the intervention?

F. Was the outcome assessor blinded to the intervention?

G. Were co-interventions avoided or similar?

H. Was the compliance acceptable in all groups?

I. Was the dropout rate described and acceptable?

J. Was the timing of the outcome assessment in both groups comparable?

K. Did the study include an intention-to-treat analysis?

### Description of study characteristics

All 7 studies included were retrospective and investigated the effect of bracing in patients with stable thoracolumbar compression fractures, nonoperatively treated (Table 3). The compression of the vertebra at admission ranged from 11% to 25%. Most fractures were located at T12 and L1. In 2 studies (Karjalainen et al. 1991, Schlickewei et al. 1991), the number of patients in the treatment group was considerably smaller than the number of patients in the control group. In the other studies the number of patients between groups was similar. The time of wearing a brace varied from 32 days to 9 months. Reported indications for bracing differed between studies and depended on the type of fracture, or they were not described at all. Co-interventions, such as start of mobilization with a brace and duration of bed rest, varied between studies and between groups within some studies. The duration of bed rest varied from 3 to 7 days. There was also heterogeneity in follow-up time, which varied between 11 months and 7 years on average.

**Table 3.** Description of study characteristics

Authors	Participants	Interventions	Follow-up mean (range)	Results
Braun et al. (1991)	n=112 Stable thoracolumbar compression fractures, conservatively treated.	Intervention: 4 days bed rest followed by mobilization with 3 point corset (n=55). Control: 4 days bed rest; mobilization without 3 point corset (n=50).	11 months	Outcome measures: Subjective complaints on an ordinal scale range 0 to 3 (0= no complaints till 3= much complaints with restriction of activities). Narrowing of the vertebra on radiography. Scoliosis, pain by pressure and muscle tension on clinical examination, not further specified. Results: Subjective complaints grading: Intervention: 1.0 Control: 0.6. Narrowing of the vertebra: Intervention: 6% Control: 8%. Scoliosis, pain by pressure and muscle tension: Separate group values for the intervention and control group were not reported. Complications: Intervention: not reported, Control: not reported.
Folman and Gepstein (2003)	n=85 Stable wedge- type compression fractures in the thoracolumbar and lumbar region (T11–L2, mostly T12), conservatively treated.	Intervention: 10 days bed rest followed by mobilization with 3 point corset for a mean period of 32 days (n=41). Control: 10 days bed rest followed by mobilization without 3 point corset (n=44).	9 years (3–16)	Outcome measures: Pain on an ordinal scale range 1 to 10. Overall disability score on modified Oswestry scale. Results: Separate group values for the intervention and control group were not reported. No statistically significant differences were found between the groups on the severity of back pain or subsequent disability. Complications: Intervention: not reported, Control: not reported.
Karjalainen et al. (1991)	n=126 Stable compression fractures of the thoracolumbar (56%) or lumbar spine, conservatively treated.	Intervention: 6 days bed rest followed by mobilization with a extension brace for a mean period of 6 weeks (n=21). Control: 3 days bed rest followed by mobilization without an extension brace (n=105).	7 years (5.5–11)	Outcome measures: Vertebral deformity (% compression, Gibbus and scoliosis angles, measured as described by Cobb) on radiography. Functional outcome classified as good (no long-term backache, no disability award, early return to work after 3-5 months and light occasional pain on exertion) or poor (persistent of severe pain on exertion, occasional pain at rest, absence from work more than six months, disability award, less physically stressful work or retirement) Results: Compression: Intervention: on admission: 25%, at late control: 32%. Control: on admission: 21%, at late control: 27%. No statistically significant difference was found between the groups. Gibbus /scoliosis angle: Intervention: on admission: 12°/3.9°, at late control: 17°/3.9°. Control: on admission: 9.9°/2.3°, at late control: 13°/3.0°. No statistically significant difference was found between the groups. Poor functional outcome: Intervention: 19% Control: 17%. No statistically significant difference was found between the groups. Complications: Intervention: not reported, Control: not reported.
Ohana et al. (2000)	n=129 Stable thoraco- lumbar and lumbar fractures from T12 to L5 (mostly L1), graded as Frenkel A, conservatively treated.	Intervention: Early mobilization with a brace (n=71) Control: Bed rest followed by late mobilization without a brace (n=58)	12 months after injury	Outcome measures: Vertebral deformity (% compression and Gibbus angle, measured as described by Cobb) on radiography. Results: Compression: Intervention: on admission: 19%, one year later: 15% (range 5%–30%). Control: on admission: 11%, one year later: 11% (range 5%–30%). No statistically significant difference was found between the groups. Gibbus angle: Intervention: on admission: 9.7°, one year later 9.6° (range 5°–25°). Control: on admission: 5.7°, one year later 5.7° (range 5°–20°). No statistically significant difference was found between the groups. Complications: Intervention: not reported, Control: not reported.
Post et al. (2006)	n=33 Stable Thoracolumbar and lumbar fractures (T10-L4, mostly T12/L1), type A 1.1 of A1.2 (Comprehensive Classification (Magerl et al. 1994)) conservatively treated	Intervention: A2, A3 and more severe A1.2 fractures: 2-6 weeks bed rest followed by mobilization with a three point brace for a mean period of 9 months (n=18) Control: A1.1 and A1.2 fractures: mobilization without a brace (n=15)	5 years (3–8)	Outcome measures: Maximum lifted load (%norm value) evaluated with Dynamic lifting test. Restrictions in activities due to back pain assessed with the Roland Morris Disability Questionnaire (RMDQ, score range 0–24, with lower scores indicating less restrictions) and with the Visual Analogue Scale Spine Score (VAS score range 0-100, with higher scores indicating better results); Quality of life was assessed with the RAND Short Form-36. Return to work status was evaluated. Results: Dynamic lifting test: Intervention: Mean 1.9 LD, SD 0.9 (range 0.3–2.7); 40% of patients scored below norm. Control: Mean 2.0 LD, SD 0.7 (range 0.9–2.7); 33% of patients scored below norm. No significant difference was found between the groups (p=0.8). RMDQ: Intervention: Mean 4.4 SD 5.5 (range 0–17). Control: Mean 6.1 SD 6.4 (range 0–17). No significant difference was found between the groups (p=0.4). VAS: Intervention: Mean 82 SD 19 (range 39-100). Control: Mean 75 SD 19 (range 36–97). No significant difference was found between the groups (p=0.2). SF-36: In the nine sub-scales no statistically significant differences were found between the braced and unbraced groups. Return to work status: Separate group values were not reported. Complications: Intervention: not reported, Control: not reported.
Schlinkewei et al. (1991)	n=124 Stable compression fractures, conservatively treated.	Intervention: 7 days bed rest followed by mobilization with a three point corset (n=102). Control: 5 days bed rest followed by mobilization without a three point corset (n=22)	2.5 years	Outcome measures: Subjective judgment of treatment effect (very good, good, moderate, bad). Gibbus angle and scoliosis on radiography. Judgment on clinical and radiographic outcome (very good, good, moderate, bad). Results (n=87): Subjective judgment: Intervention: very good: 32%, good: 40%, moderate: 22%, bad:1%. Control: very good: 27%, good: 40%, moderate: 33%, bad: 0%. Gibbus angle and scoliosis: Separate group values for the intervention and control group were not reported. Judgment on clinical and radiographic outcome : Intervention: very good: 26%, good: 60%, moderate: 14%, bad: 0%. Control: very good: 20%, good: 73%, moderate: 7%, bad: 0%. Complications: Intervention: not reported, Control: not reported.
Shen and Shen (1999)	n=38 patients stable burst fractures of T11, T12- L1 (mostly), or L2, neurological intact, conservatively treated.	Intervention: treated with Jewett brace (n=9) Control: treated without brace (n=29)	4 years (2–6)	Outcome measures:Kyphosis on radiography. Pain and return to work status on the scale of Denis et al. (P1= no pain to P5= severe pain, W1= return to heavy labor to W5= completely disabled). LOS (Length of hospital stay). Results: Separate group values for the intervention and control group of any of the outcome measures were not reported. Complications: Intervention: none reported, Control: not reported.

There was wide variation in outcome measures. 1 study reported radiographic outcome only (Ohana et al. 2000). 2 studies included only clinical outcome (Folman and Gepstein 2003, Post et al. 2006). The 4 other studies included measured both radiographic and clinical outcome (Braun et al. 1991, Karjalainen et al. 1991, Schlickewei et al. 1991, Shen and Shen 1999). Significant effects of bracing were not found in any of the studies included.

The 10 case series without a control group that were studied showed similar radiographic and functional results. Increase in kyphosis varied from 3 to 5 (mean 4), compression varied from 26% to 30% (mean 28%). Satisfactory pain and work scores were found to be less than or equal to P3 and W2 according to the pain and work scales of Denis et al. (1984).

### Data analysis

Due to the low methodological quality of the studies, no best-evidence synthesis could be performed.

### Incidence of complications

Only Shen and Shen (1999) addressed the problem of pressure ulcers and found none. Furthermore, an observational study without a control group reported a complication of bracing (Tezer et al. 2005). Of 48 patients with a thoracolumbar fracture who were non-operatively treated with a brace, 2 experienced skin problems that were not properly specified. Other complications such as emotional distress were not reported.

## Discussion

Based on the current literature, we found no evidence for the effectiveness of bracing in patients with traumatic thoracolumbar fractures, whether nonoperatively or operatively treated.

The studies we identified were all retrospective, and described patient series with thoracolumbar fractures treated with or without a brace. None of these studies fulfilled 50% or more of the internal validity criteria. Because of the incomplete methodological description of the studies, it was difficult to assess whether the methodological quality itself or only the description was insufficient. Indications for bracing were not explicitly described in most studies. Confounding by indication and selection bias seems very likely. Other possible co-interventions, such as the use of medication or physiotherapy, were not described. Although most studies included clinical outcome measures, the main focus was on radiolographic outcome. In our opinion, exclusively radiolographic evaluation does not seem adequate for assessment of the usefulness of bracing.

None of the studies selected compared bracing with non-bracing in patients with unstable thoracolumbar fractures. Several observational studies without a control group (Blamoutier et al. 1992, Chow et al. 1996) described patients with unstable fractures, nonoperatively or operatively treated with additional bracing. We did not find any studies reporting on unstable thoracolumbar fractures that were nonoperatively treated without a brace. In 6 of the 7 studies included, the incidence of complications of wearing braces was not mentioned. Other series of nonoperatively treated patients with brace have shown complications such as decubitus, but these reports contain a heterogeneity of patients also with neurological deficits (Hartman et al. 1995). In the studies and case series included, no serious complications such as neurological deficit were reported in patients who were treated without a brace.

There is a need for randomized controlled trials with sufficient sample size to allow detection of clinically relevant differences. It is important that the methodological quality of RCTs is well described, to avoid potential bias in selection, performance, exclusion, and detection.

It is obviously difficult to blind patients and care providers as to treatment. It is therefore important to achieve adequate concealment of treatment allocation. Apart from radiological and functional outcome, it is also relevant that patient-centered outcome should be measured, such as pain, anxiety, activity status, return to work, and quality of life. Complications, co-interventions, and dropout rate should be adequately reported. Although none of the studies included in our review mentioned this problem, many patients with thoracolumbar fractures have psychiatric and social problems (Matsunaga et al. 2005, Siebenga et al. 2006). Studies could be seriously hampered by this co-morbidity. Thus, compliance to treatment should also be recorded. Long-term follow-up and intention-to-treat analysis are strongly recommended. Inclusion of an economic evaluation in such trials would also be useful.

The value of bracing in patients with stable and unstable thoracolumbar fractures remains unclear. We recommend a careful and critical approach in the decision making, taking into consideration (on a case-to-case basis) patient benefits, burden, and cost of care (“weak recommendation” based on low- to very low-quality evidence according to the grading system of quality of evidence and strength of recommendations (Schunemann et al. 2006)).
